# Height and skeletal morphology in relation to modern life style

**DOI:** 10.1186/s40101-015-0080-4

**Published:** 2015-12-08

**Authors:** Michael Hermanussen, Christiane Scheffler, Detlef Groth, Christian Aßmann

**Affiliations:** Aschauhof 3, 24340 Altenhof, Germany; Institute of Biochemistry and Biology, University of Potsdam, 14469 Potsdam, Germany; Institute of Biochemistry and Biology, University of Potsdam, 14476 Potsdam-Golm, Germany; Chair of Statistics and Econometrics, Otto-Friedrich-University, 96045 Bamberg, Germany

**Keywords:** Pelvic breadth, Elbow breadth, Growth, Adult height, Community effect in height

## Abstract

Height and skeletal morphology strongly relate to life style. Parallel to the decrease in physical activity and locomotion, modern people are slimmer in skeletal proportions. In German children and adolescents, elbow breadth and particularly relative pelvic breadth (50th centile of bicristal distance divided by body height) have significantly decreased in recent years. Even more evident than the changes in pelvic morphology are the rapid changes in body height in most modern countries since the end-19th and particularly since the mid-20th century. Modern Japanese mature earlier; the age at take-off (ATO, the age at which the adolescent growth spurt starts) decreases, and they are taller at all ages. Preece-Baines modelling of six national samples of Japanese children and adolescents, surveyed between 1955 and 2000, shows that this gain in height is largely an adolescent trend, whereas height at take-off (HTO) increased by less than 3 cm since 1955; adolescent growth (height gain between ATO and adult age) increased by 6 cm. The effect of globalization on the modern post-war Japanese society (“community effect in height”) on adolescent growth is discussed.

## Background

We are used to long-term evolutionary changes in height and skeletal morphology, but significant changes also occur within a few decades and strongly relate to modern life style. Anecdotal evidence of immediate remodelling in muscular and skeletal built related to changes in locomotion was published by E.J. Slijper [1]. The Dutch morphologist reported on a little goat born without forelegs. For the first 7 months of its life, it passed its days on the grass-field, moving forward by jumps on its hindlegs in a semi-upright posture. The body made an angle of nearly 45° with the ground and the hoofs of the hindlegs placed much farther forward under the body than in a normal goat. The manner of locomotion was quite similar to that of a jumping-hare or a kangaroo, both hindlegs leaving the ground at the same time. Slijper later studied the anatomy of this animal and observed an elongation of the first phalanx; he found very marked increases in thickness of all bones of the hindleg and especially increases of their proximal and distal ends and joint surfaces. The greater part of the muscles of the bipedal goat was better developed than in quadrupedal goats. All the pelvic bones, especially of the acetabulum and pubis, significantly thickened due to the increase of the weight supported by the pelvis. The iliolumbar angle decreased by 23°, while the lumbo-sacral angle increased by 16°.

Remodelling in muscular and skeletal built is not restricted to bipedal goats. The comforts of modern human life are manifold. Most modern people are physically inactive; they connect with friends and relatives virtually via electronic media; they drive cars, use elevators, chauffeur their children to kindergartens and schools, and distances of up to 30 km per day, as our ancestors did, are no longer walked by modern children and adolescents. Only 13.1 % of German girls and 17.4 % of German boys are physically active for 60 min per day [2].

## Review

Parallel to the decrease in physical activity changes in skeletal measures occurred in the recent years. Rietsch et al. [3] documented an association between physical activity and relative elbow breadth in children. The finding is astonishing as elbow breadth is not that skeletal structure that one first associates with walking. But the coordinated pendulousness of shoulder and arms in fact significantly contributes to balanced walking [3, 4] and might explain this association. Much greater than the effects on elbow breadth, however, are the effects of bipedal locomotion on the pelvic bones.

**Fig. 1:**
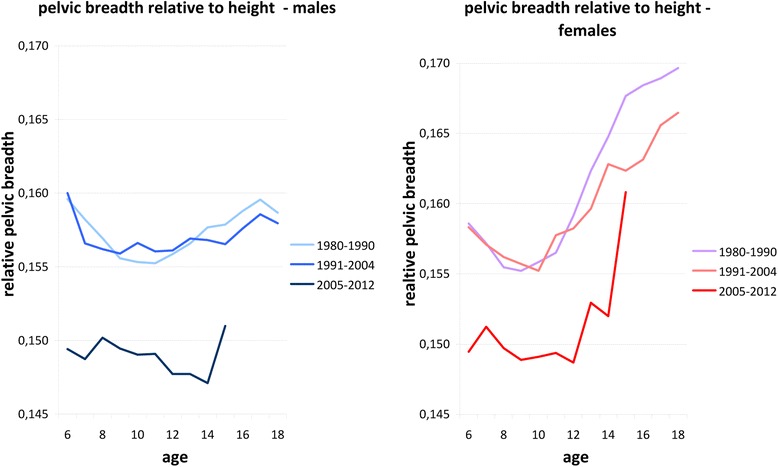
Relative pelvic breadth (50th centile of bicristal distance divided by body height). The 2005–2012 cohorts were significantly slimmer than the cohorts investigated in 1980–1990 and 1991–2004 [5]

**Fig. 2:**
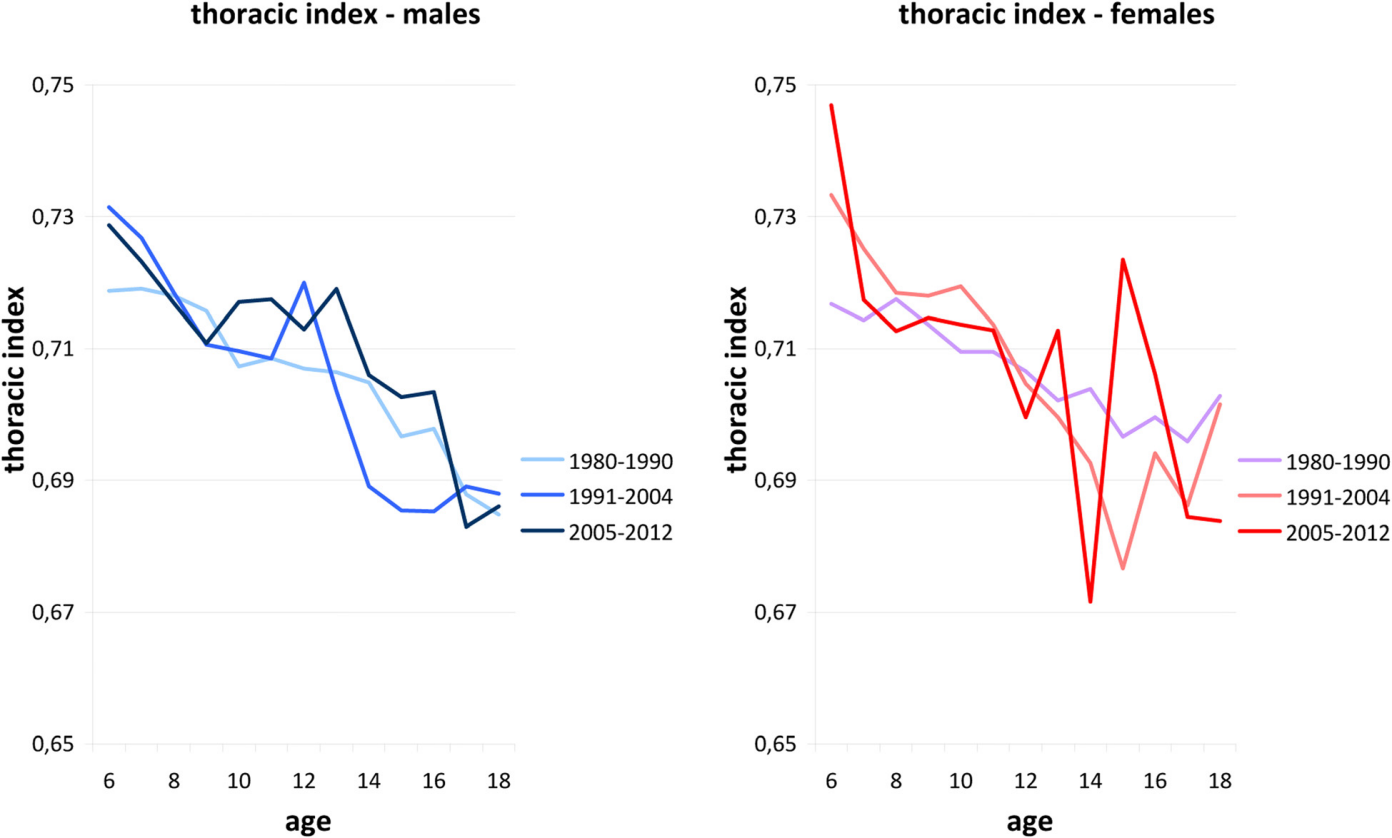
Fiftieth centiles of thorax index (thoracic breadth / thoracic depth). There was no evidence of slimming in the thorax [5]

Anthropometric data of height, bicristal pelvic breadth, and thoracic index (thorax index = thoracic breadth / thoracic depth) were re-investigated in up to 28,975 healthy females and up to 28,288 healthy males, aged 3–18 years [5] from 10 cross-sectional surveys. The data had been obtained by members of the anthropological departments of the Universities of Potsdam and Berlin, Germany [3, 6–10], between 1980 and 2012. The data were divided into three time slots (1980–1990, 1991–2004, and 2005–2012). Figure 1 illustrates relative pelvic breadth (50th centile of bicristal distance divided by body height). The 2005–2012 cohorts were significant slimmer than the cohorts investigated in 1980–1990 and 1991–2004 [5]. Skeletal measures of the thorax were used for control as this part of the body is not involved in bipedal locomotion. There was however no evidence of slimming in the thorax (Fig. 2).

**Fig. 3:**
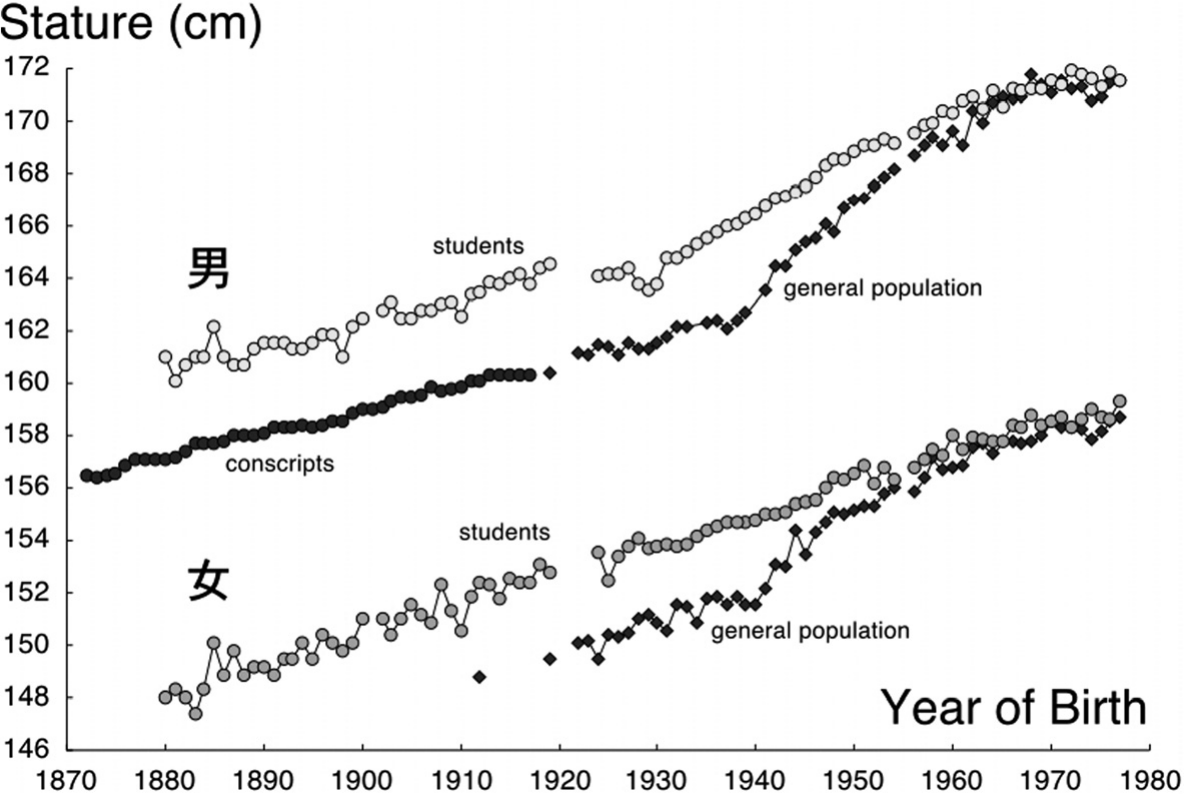
Body height of Japanese man and women, born since 1870 [17]. The data are reproduced from the original publication with kind permission of the Anthropological Society of Nippon©

Even more evident than the changes in pelvic morphology are the rapid changes in body height in most modern countries since the end-19th and particularly since the mid-20th century. Figure 3 shows the Japanese trend. Japanese slightly increased in height since the end-19th century and rapidly increased since the mid-20th century. Yet when looking more closely, the figure is amazing. There is some common agreement that stunted children may not be able to fully reach their genetic target in body height [11]. In this light, we might expect Japanese born in 1939 and shortly thereafter who had experienced the disastrous living conditions of the war and post-war period to be stunted. But this is not the case. The early war-born cohorts were those cohorts who started the exceptional trend towards taller stature.

The data suggest that adult height rather reflects living conditions during adolescence than the situation during infancy and early childhood. Similarly rapid trends in height were observed in adolescent Germans. Before 1990, East Germans used to be shorter than West Germans. In 1990, the socialistic East and the capitalistic West German states re-unified. Thereafter, it took just 4 years for East German military conscripts to completely catch-up the 2.5-cm difference that formerly existed between them and their West Germans peers [12]. Similar mechanism may have prompted the war-born Japanese boys who grew up in the mid-1950s among the tall and victorious American GIs.

The authors have proposed a “community effect in height” [13]. It appears that tall societies generate tall people, and short societies generate short people. The hypothesis suggests that body height in the immediate vicinity of an adolescent serves as target when regulating his/her final height. The hypothesis is based on observations of height distributions from historical 19th and 20th century records of military conscripts. Body height tends to cluster. In the 19th century, practically all European people were short. Today, practically all people are tall. There is little overlap between historic and modern height distributions. For example, in 1863, the average Dutch conscript reached 165 cm, and less than 1 % of these conscripts reached the mean body height of modern Dutch men of 184 cm. Thirty percent of the historic conscripts failed to reach 157 cm, which is less than the 1st centile of the modern Dutch growth charts.

Using a Bayesian modelling approach and data from a longitudinal study of school children and adolescents from Zurich, Switzerland, the authors analysed the final period of growth and found that in addition to well-known predictors of adult height, such as bone age and Tanner stages of puberty, there is evidence for a new parameter that they define as “past relative height.” This parameter operates during the adolescent growth period to adjust the growth rate of an individual towards the average height of her/his immediate community. It appears that the smaller the adolescent is compared with past mean average height of the community, the more the adolescent grows during puberty. Conversely, taller than average adolescents will grow less. The net outcome is that the distribution of heights of members of a community (or a social network) will cluster towards the mean value.

Empirical support for this community effect on height comes from the analysis of Swiss conscripts measured in 1884–1891, in 1908–1910, and in 2004–2009 [14]. Mean district heights were calculated for each of the 169 districts (political subdivisions of Switzerland’s cantons). Mean district heights were then correlated by distance between the districts (*p* < 0.01). Random network analyses suggested a direct road effect on height—closer distance by road and not “as the crow flies” direct distance—resulted in a greater correlation in height between districts. The analyses controlled statistically for income variation and for iodine deficiency (goitre prevalence) between districts, suggesting that the spatial association of body height among the Swiss conscripts is incompletely explained by wealth or health. The data suggest that people may simply be short because their friends and neighbours are short or tall because their friends and neighbours are tall. This is the community effect on height, due to, perhaps, psycho-biological effects on growth and development within networks of people who directly or indirectly interact with each other because they are linked by roads. First studies in IGF suggest that this hormone may play an important role in this regulation [15].

**Fig. 4:**
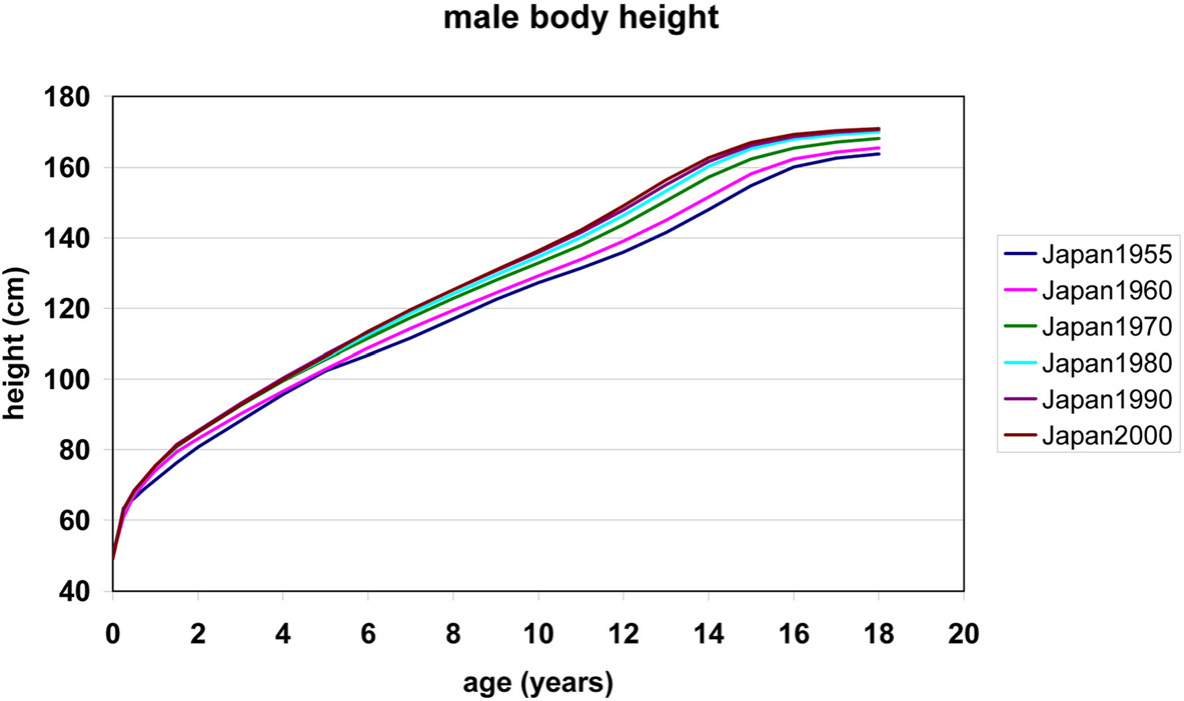
Body height in six national Japanese surveys [18, 19]

The community effect in height regulates growth during adolescence. Also, the secular trend in height appears to be an adolescent trend. Preece-Baines modelling of six national samples of Japanese children and adolescents, surveyed between 1955 and 2000, highlights this phenomenon. Preece-Baines modelling [16] is particularly suitable to distinguish between the childhood/preadolescent and the adolescent period of growth. The model defines an “age at take-off” (ATO) when the adolescent growth spurts starts. ATO thus provides an estimate of developmental tempo. ATO in fast maturing subjects or cohorts is early. Figure 4 demonstrates mean values for height in the six national Japanese surveys. Table 1 summarizes “age at take-off” and “height at take-off”. Modern Japanese mature earlier, and they are taller at all ages. It is evident that the gain in height since the mid-20th century is largely an adolescent trend. Whereas height at take-off increased by less than 3 cm since 1955, adolescent growth (height gain between age at take-off and adult age) increased by 6 cm. We are inclined to attribute the rapid height gain of the young generation since 1955 to a new “cosmopolitan community target” that has established in an open-minded and modern post-war Japanese society.

**Table 1:** “Age at take-off (ATO)”, “height at take-off (HTO)”, adult height, and adolescent growth [16] in six national Japanese surveys published between 1955 and 2000 [17, 18]

	ATO	HTO	Adult height	Adolescent growth
	Years	cm	cm	cm
Japan 2000	8.1	125.39	171.05	45.66
Japan 1990	8.3	126.30	170.47	44.17
Japan 1980	8.3	125.13	169.77	44.64
Japan 1970	8.7	126.03	167.53	41.50
Japan 1960	9.1	124.39	165.39	41.00
Japan 1955	9.3	122.92	162.64	39.72

## Conclusions

Height and skeletal morphology strongly relate to life style. Both the changes in physical exercise and in the environmental, psycho-social, and possibly even emotional, changes during the last decades have left significant imprints in modern skeletal built.
